# Importance of Primary Healthcare in prognosis of individuals with *diabetes mellitus* and/or hypertension

**DOI:** 10.31744/einstein_journal/2021CE6773

**Published:** 2021-12-14

**Authors:** Aline Rodrigues de Paiva

**Affiliations:** 1 Centro Universitário Alfredo Nasser Goiânia GO Brazil Centro Universitário Alfredo Nasser, Goiânia, GO, Brazil.

Dear Editor

I bring considerations as to the article, “Profile of patients with hypertension and/or *diabetes mellitus* in Primary Healthcare units.”^( 1 )^ The importance of prevention and health promotion when related to chronic diseases is well known, making Primary Healthcare decisive and categorical, since it performs basic interventions involving harm reduction and health maintenance. Within the group of chronic diseases,^( 2 )^ *diabetes mellitus* and hypertension currently account for the main causes of mortality, a major global public health problem.^( 3 )^

Thus, aggregating and updating the epidemiological data cited in the article highlight the importance of discussing Primary Healthcare in relation to the diseases in question. In 2019, one in every 11 people aged between 20 and 79 years were affected by *diabetes mellitus* and hypertension in the world, resulting in 463 million individuals diagnosed, with a profile of higher prevalence among women and adults over 65 years of age.^( 4 , 5 )^

The perspective is that this figure will increase by 51% by the year 2045, which means 700 million individuals with *diabetes mellitus.* Due to the lack of access to information and public health, 50% of individuals with *diabetes mellitus* have not been identified yet, leading to the development of severe complications, which generate overload and high hospital costs, in addition to worsening the prognosis and quality of life of the individual.^( 5 )^

I congratulate the authors for publishing this study, which should be extensively discussed and prioritized to debate the theme in the academic and care settings, ensuring that Primary Health Care achieves excellence!
