# Supplementary material for: Importance of Primary Healthcare in prognosis of individuals with diabetes mellitus and/or hypertension
Source: Einstein (Sao Paulo). 2021 Dec 14;19:eCE6773. doi: 10.31744/einstein_journal/2021CE6773 (PMC8687647; doi:10.31744/einstein_journal/2021CE6773)
Supplement: Translation

# Translation: A importância da Atenção Primária no prognóstico de indivíduos com *diabetes mellitus* e/ou hipertensão arterial

**Authors:** Aline Rodrigues de Paiva

**Affiliations:** 1 Goiânia GO Brasil Centro Universitário Alfredo Nasser, Goiânia, GO, Brasil.

Caro Editor,

Trago considerações acerca do artigo “Perfil de pacientes com hipertensão arterial e/ou *diabetes mellitus* de unidades de Atenção Primária à Saúde”.^( 1 )^É conhecida a importância da prevenção e da promoção à saúde quando relacionadas às doenças crônicas, tornando a Atenção Primária à Saúde decisiva e categórica, visto que ela realiza intervenções básicas, que envolvem redução de danos e manutenção da saúde.^( 2 )^O *diabetes mellitus* e a hipertensão arterial atualmente são responsabilizadas, dentro do grupo de doenças crônicas, como principais causas de mortalidade, enquadrando-se como um grande problema de saúde pública global.^( 3 )^

Assim, agregar e atualizar os dados epidemiológicos citados no artigo relevam a importância de se discutir a Atenção Primária em relação às doenças em questão. No ano de 2019, uma em cada 11 pessoas entre 20 e 79 anos foram acometidas pela *diabetes mellitus* e hipertensão arterial no mundo, o que resulta em 463 milhões de indivíduos diagnosticados, com perfil de maior prevalência entre mulheres e adultos acima de 65 anos de idade.^( 4 , 5 )^

A perspectiva é que esse valor tenha aumento de 51% até o ano de 2045, o que significa 700 milhões de indivíduos com *diabetes mellitus.* Devido à falta de acesso à informação e à saúde pública, 50% dos indivíduos portadores de *diabetes mellitus* ainda não foram identificados, o que acarreta no desenvolvimento de complicações graves, as quais geram sobrecarga e alto custo hospitalar, além de piorararem o prognóstico e a qualidade de vida do indivíduo.^( 5 )^

Cumprimento os autores por publicarem este estudo, que deve ser extensivamente discutido e priorizado para debater o tema no meio acadêmico e assistencial, garantindo que a Atenção Primária alcance a excelência!
